# Analysis of the Biopsychosocial Impacts Associated with Endometriosis to Improve Patient Care

**DOI:** 10.3390/jcm14072158

**Published:** 2025-03-21

**Authors:** Aniela Roxana Noditi, Ioana-Stefania Bostan, Francesca Scurtu, Diana Ionescu, Andra Maria Mehedintu, Aida Petca, Claudia Mehedintu, Marinela Bostan, Ana Maria Rotaru

**Affiliations:** 1 Faculty of Medicine, University of Medicine and Pharmacy Carol Davila, 050471 Bucharest, Romania; aniela.noditi@umfcd.ro (A.R.N.); francesca.scurtu@umfcd.ro (F.S.); andra-maria.mehedintu0720@stud.umfcd.ro (A.M.M.); aida.petca@umfcd.ro (A.P.); claudia.mehedintu@umfcd.ro (C.M.); ana-maria.rotaru@drd.umfcd.ro (A.M.R.); 2 Filantropia Clinical Hospital, 011132 Bucharest, Romania; 3 Faculty of Medicine, Titu Maiorescu University, 040314 Bucharest, Romania; diana.ionescu@spitalgomoiu.ro; 4 ‘Stefan S. Nicolau’ Institute of Virology, Center of Immunology, Romanian Academy, 030304 Bucharest, Romania; 5 ‘Victor Babes’ National Institute of Pathology, Department of Immunology, 050096 Bucharest, Romania

**Keywords:** endometriosis, quality of life, mental health, management, reproductive health

## Abstract

Endometriosis is a non-malignant, inflammatory condition that impacts individuals across various hormonal stages, including before their first menstruation, throughout their reproductive years, and after menopause. This condition arises when tissue resembling the uterine lining grows outside the uterus, resulting in inflammation and a range of symptoms, such as dysmenorrhea, pain during intercourse, chronic discomfort, and challenges with fertility. This review provides a comprehensive analysis of the medical strategies implemented to address the pathology of endometriosis, highlighting its significant impact on the quality of life of the individuals affected by this condition. Endometriosis can influence various aspects of life, including physical health, emotional well-being, social interactions, and professional performance. Usually, to assess the quality of life in women with endometriosis, validated instruments, such as different questionnaire types, are used to measure the physical, psychological, social, and reproductive health impacts. To improve the quality of life of the women experiencing endometriosis, several supportive strategies are proposed. The findings underscore the necessity of managing endometriosis through a multidisciplinary approach that encompasses both medical and surgical interventions, lifestyle modifications, and psychological support.

## 1. Introduction

Endometriosis is a chronic condition that affects women of various ages globally, frequently resulting in severe pain and potential fertility issues, which can significantly compromise the overall quality of life. This condition is characterized by the presence of tissue resembling the uterine lining developing outside the uterus. Such tissue may be located on various pelvic organs, including the ovaries, fallopian tubes, peritoneum, and the external surface of the uterus, and in some instances, it may extend to more distant regions of the body [1]. Endometriosis is classified into three primary types based on the location and characteristics of the lesions: superficial peritoneal endometriosis, ovarian endometriosis (endometriomas), and deep infiltrating endometriosis (DIE) [2]. It is imperative to understand the distinct clinical implications and the varying severity associated with each type, as these factors directly influence the patient’s quality of life. Additionally, unique presentations such as adenomyosis (where endometrial tissue proliferates within the muscular wall of the uterus), extra-pelvic endometriosis (localized in areas such as the diaphragm, lungs, or liver), and scar endometriosis (emerging in surgical scars following cesarean sections or other abdominal surgeries) further illustrate the complexity and variability of this disease [3]. The hallmark features of endometriosis include chronic inflammation, pain, and potential infertility. An effective diagnosis involves a systematic approach that integrates a clinical evaluation with advanced imaging techniques, including ultrasound and magnetic resonance imaging. Additionally, minimally invasive surgical methods such as laparoscopy can be valuable tools in the diagnostic process, contributing to a more accurate and comprehensive understanding of the patient’s condition. Laparoscopy remains necessary for confirming diagnoses when imaging and clinical suspicion are inconclusive, as well as for surgical treatment, such as the removal of endometriotic lesions [4,5]. However, according to the latest ESHRE guidelines, surgical diagnosis is no longer required for diagnosing endometriosis. This guideline change aims to reduce unnecessary surgeries and their associated risks and costs, while ensuring accurate diagnosis and effective treatment for individuals with endometriosis [6].

Treatment strategies focus on alleviating symptoms and enhancing the quality of life through medical, surgical, and supportive interventions [7]. A thorough understanding of the underlying mechanisms and effective management strategies is crucial for delivering comprehensive care to individuals with endometriosis. While medical management can yield both positive and negative effects on the quality of life, an efficacious treatment regimen has the potential to significantly improve physical, emotional, and mental well-being. Nevertheless, the possible side effects and sustained demands of treatment, which may alter inflammatory, immune, or hormonal functions, can present additional challenges [8,9].

Sometimes, surgery is indicated for other reasons (e.g., to preserve fertility, severe symptoms, or large endometriomas). In any surgical procedure, it is essential to know the potential risks and side effects. The side effects associated with the surgical interventions for endometriosis may differ depending on the specific type of surgery performed, the overall health of the patient, and the severity of the endometriosis condition [10]. In cases where the patients present with significant symptoms, particularly those related to pain or fertility challenges, the potential benefits of surgical treatment may outweigh the inherent risks. Furthermore, individuals with a history of previous surgeries or those who have endured the effects of endometriosis for an extended period may consider surgery as a final option after exhausting other treatment modalities [11]

Ultimately, the decision to pursue surgical intervention for endometriosis is a highly individualized one. Patients should be comprehensively informed about the nature of the surgery, as well as the potential risks and benefits, to facilitate an informed decision-making process. Some studies indicate that surgical intervention for endometriosis can lead to substantial improvements in the quality of life for women affected by this condition. Post-surgery, patients often report a significant reduction in physical pain and discomfort, as well as an enhanced emotional outlook due to improved fertility prospects [12]. These favorable outcomes can transform the perspectives of women who undergo surgical procedures, enabling them to resume daily activities and regain a sense of control over their lives, thereby mitigating emotional distress [13,14].

A comprehensive examination of the multifaceted aspects associated with endometriosis may facilitate the development of health promotion initiatives that address mental, emotional, and sexual well-being. Furthermore, such initiatives should also consider the societal impact of endometriosis on women’s roles within the community.

This article aimed to highlight the evolution of the approach to a medical topic “endometriosis,” from diagnosis and therapeutic approach to the healing of psycho-emotional problems. In the first part, the causes of the occurrence of endometriosis, from genetic and epigenetic factors to environmental factors, are briefly described. The data presented emphasize the need to manage endometriosis with multidisciplinary approaches that integrate medical and surgical aspects, lifestyle changes, and psychological support. Thus, in order to present a complete picture of the evolution of the medical approach in resolving the pathology of endometriosis, the second part of the review addresses the topic of the impact of endometriosis on the quality of life (QoL) with multiple effects at the physical, emotional, social and professional levels. At the end of the review, several support strategies are presented that could improve the quality of life of women with endometriosis. This review supports the ongoing concerns of physicians to approach the treatment of endometriosis as effectively as possible, accompanied by an openness to knowing and integrating the impact of the endometriosis diagnosis in the patient’s life.

## 2. Endometriosis Causes

### Multifactorial Aspects of Endometriosis

The precise etiology of endometriosis remains incompletely understood; however, several theories have been formulated to elucidate its development [15]. One prominent hypothesis is known as Sampson’s Theory of Retrograde Menstruation, which proposes that a portion of menstrual blood may reverse its typical trajectory during the menstrual cycle. Instead of being expelled from the body, this blood travels through the fallopian tubes into the pelvic cavity. Consequently, endometrial cells contained within this blood may implant and proliferate outside the uterine environment [16]. Another compelling theory, referred to as Coelomic Metaplasia, suggests that certain cells within the pelvic cavity derived from the same embryonic precursors as the endometrium can differentiate into endometrial-like cells in response to various stimuli [17]. The Embryonic Cell Rest theory posits that cells resembling endometrial tissue, which are retained from the embryonic developmental phase, may persist in the pelvic cavity. Under specific physiological conditions, these cells have the potential to evolve into endometriosis [18]. Moreover, a dysfunction of the immune system may contribute to the failure in recognizing and eliminating endometrial cells located extraneously in the uterus. Anomalies within the immune system could further exacerbate the inflammation and pain associated with endometriosis [19]. An additional hypothesis suggests that endometrial cells may disseminate via the lymphatic system or bloodstream to remote anatomical sites, such as the lungs or brain. This phenomenon may provide a plausible explanation for the occurrence of endometriosis in these distant locations [20].

Endometriosis is a condition influenced by a complex interplay of genetic, immunological, hormonal, and environmental factors. A thorough understanding of these elements is essential for the development of effective diagnostic and treatment strategies for individuals affected by this disorder.

Genetic and epigenetic factors significantly contribute to the susceptibility to endometriosis [21]. A familial history of the condition, along with specific genetic variations, can heighten the risk of developing endometriosis. While the exact genetic mechanisms remain partially elucidated, researchers have identified certain genes associated with an increased risk [22]. Notably, Single Nucleotide Polymorphisms (SNPs) in the WNT4 (Wnt Family Member 4), VEZT (Vezatin, Adherens Junctions Transmembrane Protein), and CDKN2B-AS1 (Cyclin-Dependent Kinase Inhibitor 2B Antisense RNA 1, also known as ANRIL) genes have been linked to dysregulations of the immune response and hormonal secretion [23,24]. Chromosomal anomalies observed in chromosomes 1, 7, 9, and 10 have also been correlated with a greater risk of developing endometriosis. Furthermore, epigenetic alterations, such as DNA methylation and histone modifications, are critical in the development and persistence of the condition, as they affect the expression of genes relevant to endometriosis [25].

The role of inflammatory processes in the onset and progression of endometriosis is underscored by elevated levels of proinflammatory cytokines, including IL-1, IL-6, and TNF-α, in the peritoneal fluid of affected individuals. These cytokines not only facilitate inflammation but also support the viability of endometrial cells outside the uterine cavity [26,27]. Cellular-level changes associated with the immune response include an increased macrophage activation, which contributes to a chronic inflammatory environment. Additionally, perturbations in T cell functionality, characterized by a rise in regulatory T cells (Tregs) and disrupted CD4/CD8 T cell ratios, may impair the immune system’s ability to eliminate ectopic endometrial cells. The deactivation of natural killer (NK) cells, which are responsible for the elimination of aberrant cells, further facilitates the implantation and proliferation of endometrial tissue outside the uterus. Some studies indicate that endometriosis exhibits features analogous to autoimmune diseases, such as the presence of autoantibodies, which suggests that immune system dysregulation may play a role in the onset and persistence of the condition [28].

Hormonal imbalances, particularly regarding progesterone resistance and estrogen dominance, have been identified as significant factors in the development of endometriosis. The interplay among hormonal, genetic, and immunological elements is critical to understanding the pathogenesis of this condition. Endometriosis is dependent on estrogen, and genetic variations in estrogen receptors (ER-α and ER-β) as well as enzymes involved in estrogen synthesis, such as aromatase (encoded by the CYP19A1 gene), may predispose individuals to increased susceptibility and severity. Furthermore, estrogen promotes the growth of endometrial tissue, including ectopic lesions, while dysregulated expression of progesterone receptors can lead to diminished responsiveness to progesterone, allowing for unopposed estrogenic stimulation and perpetuation of endometrial lesions [29].

Environmental factors also play a significant role in the emergence and progression of endometriosis. Exposure to environmental toxins, such as dioxins and polychlorinated biphenyls (PCBs), has been suggested to increase the risk of endometriosis by disrupting hormonal and immune functions, which may contribute to the condition’s pathogenesis. [30]. Various risk factors associated with endometriosis include family or reproductive history, lifestyle factors (such as low body mass index, high caffeine or alcohol consumption, and insufficient physical activity), and medical conditions linked to uterine abnormalities and autoimmune diseases. These factors have been correlated with an elevated risk of developing endometriosis [31].

A comprehensive understanding of these multifaceted influences is imperative for devising tailored therapeutic interventions and personalized management strategies for individuals impacted by endometriosis.

## 3. Endometriosis Management

Effective management of endometriosis necessitates a multidisciplinary approach that integrates medical and surgical treatments alongside lifestyle modifications and psychological support. The severity of the symptoms significantly impacts the selection of treatment options, the extent of the disease, and the patient’s fertility objectives. The primary methods of treatment encompass pain management, hormonal therapy, surgical interventions (such as laparoscopy, laparotomy, or hysterectomy), and fertility treatments [32].

The overarching goals in the management of endometriosis include the alleviation of pain, control of disease progression, and addressing fertility concerns. It is crucial to tailor the treatment approach based on the severity of symptoms, the extent of the disease, the patient’s age, fertility considerations, and overall health status [33]. A personalized strategy that takes into account the patient’s symptoms, disease severity, and reproductive aspirations may enhance their quality of life. The implementation of a combination of medical and surgical interventions, lifestyle adjustments, and supportive therapies can effectively address both the symptoms and the fertility concerns. Regular evaluation and modification of the treatment plan are essential to achieving optimal outcomes for individuals diagnosed with endometriosis.

## 4. Endometriosis Impact on the Quality of Life

Endometriosis represents a significant health concern for women globally, affecting individuals from adolescence, through their reproductive years, and extending to the peri- and post-menopausal stages [34]. This condition can markedly influence work productivity and overall quality of life [35]. Symptoms associated with endometriosis—including chronic pelvic pain, dysmenorrhea, menorrhagia, dysuria, dyspareunia, and dyschezia [36]—can severely impair women’s quality of life. Furthermore, endometriosis has a profound impact on the quality of life (QoL) for those affected, influencing multiple dimensions of physical, emotional, social, and professional well-being [37].

### 4.1. Physical Impact of Endometriosis on Women

Comparative studies indicate that endometriosis exerts a significant impact on women’s physical health, highlighting several critical aspects, including chronic pain, reduced physical functioning, infertility, and complications related to menstrual and sexual health [38,39,40]. This condition profoundly affects multiple dimensions of women’s lives, often presenting as persistent pelvic pain that is not confined to menstrual cycles, thereby influencing intimate relationships [41]. Endometriosis may lead to confusion with urinary tract infections, as it can involve painful urination. Additionally, individuals may experience severe pain during bowel movements, which can adversely affect digestive health. Excessive menstrual bleeding has the potential to result in anemia and the related fatigue, while irregular menstrual cycles complicate daily planning and contribute to increased stress levels. Symptoms such as frequent bloating, nausea, and digestive disturbances may mimic or co-occur with irritable bowel syndrome (IBS) [42]. Furthermore, endometriosis can lead to structural abnormalities and inflammation that obstruct conception and pregnancy. Hormonal treatments utilized for managing this condition may induce side effects, including weight gain, mood fluctuations, and diminished bone density. The chronic pain associated with endometriosis can restrict physical activity and exercise, thereby resulting in decreased overall physical fitness and additional weight gain [43]. The debilitating physical symptoms of endometriosis significantly hinder daily activities, occupational performance, and social interactions.

### 4.2. Emotional Impact of Endometriosis on Women

The chronic nature of endometriosis, combined with its physical manifestations and potential implications for fertility, can result in a range of emotional challenges. Numerous studies have demonstrated that a diagnosis of endometriosis significantly affects the emotional well-being of women [44,45,46]. Persistent pain contributes to a state of ongoing stress, anxiety, and frustration. The unpredictability of pain episodes can induce feelings of helplessness and a perceived loss of control over one’s life. The emotional and physical burdens of managing endometriosis can also influence family relationships, often leading to misunderstandings or a lack of support. Difficulties in conceiving can create profound distress and may exacerbate ongoing tensions within familial dynamics [47]. Furthermore, the emotional repercussions of infertility treatments, coupled with the uncertainty surrounding their outcomes, can intensify these feelings. Additionally, the constraints imposed by endometriosis may diminish self-esteem and confidence, thereby complicating efforts to maintain a positive self-image. It is evident that the emotional ramifications of endometriosis on women’s lives are significant and multidimensional. The continual pain, the challenges related to fertility, the stress of daily management, and the psychological burden of treatments can culminate in depression, anxiety, and a reduced quality of life [48]. Recognizing and addressing these emotional challenges is imperative for delivering comprehensive care to women affected by endometriosis.

### 4.3. Mental Well-Being Impact

Numerous studies have identified distinct correlations between the factors associated with endometriosis and the mental well-being of women diagnosed with this condition. Following the diagnosis, many women exhibit elevated levels of anxiety, particularly in relation to the severity of pelvic pain [49,50,51]. Furthermore, mental health outcomes appear to be significantly influenced by individual variables such as self-esteem, body image, and emotional self-regulation [4]. Several investigations have sought to establish a connection between endometriosis and psychological disorders. The findings provide substantial evidence supporting the hypothesis that endometriosis is linked to a range of psychiatric symptoms, especially depression, anxiety, and psychosocial stress, which ultimately result in a reduced quality of life [52,53,54]. Notably, the study conducted by Sepulcri R. found that over 80% of participants demonstrated symptoms of depression and anxiety, irrespective of the severity of their endometriosis [55,56,57]. Additionally, Cavaggioni G.’s research suggests that the data may be influenced by the higher prevalence of alexithymia in individuals with endometriosis, complicating the identification of mental disorders due to difficulties in recognizing and articulating emotions [58]. The psychological state of women with endometriosis may also be adversely affected by concurrent chronic pain conditions, such as migraines or spinal pain [59,60]. A study by Surrey ES further indicated a markedly higher incidence of comorbidities—such as ovarian cysts, uterine fibroids, and infertility among women diagnosed with endometriosis compared to those without the condition [61].

### 4.4. Endometriosis Impact on Daily Life—Social and Professional

Endometriosis significantly affects various dimensions of a woman’s daily life, notably within social and professional contexts [62]. The chronic nature of this condition, compounded by symptoms such as persistent pain, fatigue, and emotional distress, can severely impede an individual’s capacity to participate in everyday activities and maintain interpersonal relationships. A multitude of studies has examined the emotional ramifications of endometriosis, indicating that women diagnosed with this condition frequently experience disconnection from social networks, resulting in feelings of isolation and loneliness [63,64]. Furthermore, dyspareunia—painful intercourse—can exert considerable strain on intimate relationships, leading to feelings of frustration, guilt, and diminished intimacy. Partners may encounter challenges in comprehending the complexities of the condition, which can precipitate communication breakdowns. The emotional and physical demands of managing endometriosis can also permeate family dynamics, potentially resulting in stress and misunderstandings among family members. In cases where infertility arises, individuals may experience heightened anxiety and depression, complicating social interactions and leading to withdrawal from social engagements [65].

Endometriosis is a chronic inflammatory condition that can significantly impact sleep quality due to pain, hormonal imbalances, and emotional distress. Many women with endometriosis report insomnia, poor sleep quality, excessive daytime fatigue, and difficulty staying asleep [66].

Professionally, the diagnosis of endometriosis can adversely impact performance and productivity. The chronic pain and fatigue typically associated with this condition often result in frequent absenteeism, thereby affecting job performance, opportunities for career advancement, and overall job security. The symptoms can hinder concentration and task execution, ultimately diminishing productivity and work quality [67]. Additionally, regular medical appointments, surgical interventions, and requisite recovery periods can lead to missed professional opportunities, promotions, or critical engagements. The financial implications of managing endometriosis—including expenses related to medications, surgeries, and complementary therapies—can be substantial. In severe instances, some individuals may be compelled to reduce their working hours or even terminate their employment, resulting in diminished income and loss of financial autonomy. This economic strain can further exacerbate stress and influence long-term financial stability [68].

### 4.5. The Impact of Lifestyle Factors on the Quality of Life in Endometriosis

Lifestyle factors such as alcohol, smoking, diet (red meat, protein, fiber), and vitamins can significantly affect symptoms and overall quality of life. Alcohol consumption can affect liver function, increase estrogen levels, and contribute to systemic inflammation, worsening pain, and fatigue. Also, the negative effects induced by smoking are generated by reducing oxygenation and the occurrence of a hormonal imbalance by modulating the amount of estrogen or by increasing oxidative stress that amplifies the inflammatory processes. Some research indicates that women who engage in high alcohol consumption, smoke, or have a high intake of red meat may have a higher risk of developing endometriosis, which is associated with more severe pain and discomfort [69]. On the other hand, it is known that diets rich in fiber reduce estrogen levels and inflammation, improving quality of life and reducing pain in women with endometriosis. Furthermore, certain vitamins and nutritional supplements have demonstrated beneficial effects on immune system regulation, inflammation reduction, and oxidative stress mitigation, thus reducing the pain associated with endometriosis. Some studies show that women who take supplements with vitamin D, B6, magnesium, and Omega-3 often report a decrease in pelvic pain and an overall improvement in well-being [70,71]. Conversely, evidence suggests that women who engage in high alcohol consumption, smoking, or excessive red meat intake may have a higher risk of developing endometriosis, which is associated with more severe pain and discomfort [72].

## 5. Methods for Assessing the Impact of Endometriosis on QoL

For selecting the articles of interest, we predefined inclusion criteria such as studies involving human participants, studies focusing on the diagnosis, treatment, or epidemiology of endometriosis, and the impact of endometriosis on patients’ quality of life. In order to ensure the selection of pertinent articles from the PubMed and Web of Science databases, a comprehensive search strategy was implemented using the following keywords: endometriosis, quality of life, anxiety, depression, mental health, stress, emotional well-being, pain, dysmenorrhea, dyspareunia, social functioning, fatigue, productivity, and work. Only articles published in English with full-text accessibility from the years 2010 to 2024 were considered for inclusion. The review encompassed randomized controlled trials (RCTs), cohort studies, and case–control studies. Conversely, studies conducted on animal models, those lacking assessments of quality of life, conference abstracts, research focusing solely on infertility, and studies characterized by small sample sizes were excluded from the review. Ultimately, a total of 89 articles that fulfilled the established criteria were selected and incorporated into the systematic review.

Analyzing the impact of endometriosis on QoL requires methodologies that can capture the various effects this condition has on physical, emotional, social, and psychological well-being. Currently, standardized quantitative questionnaires such as the SF-36 (Short Form Health Survey) are used to assess overall health, including physical and mental health domains [73]. Another tool, the WHOQOL-BREF (World Health Organization Quality of Life-BREF), evaluates physical health, psychological health, social relationships, and environmental factors. Additionally, some disease-specific instruments are designed to assess endometriosis, such as the Endometriosis Health Profile-30 (EHP-30), which focuses on issues related to endometriosis, including pain, control, emotions, and social interactions [74,75]. Another tool is the Endometriosis Impact Questionnaire (EIQ), which evaluates the physical and emotional burden of the disease on daily life.

Various scales are commonly used to assess pain and symptoms. For example, the Visual Analogue Scale (VAS) measures the severity of pain, while the Numerical Rating Scale (NRS) quantifies pain intensity [76,77]. Additionally, the Biberoglu and Behrman (B&B) Scale evaluates symptoms such as dysmenorrhea (painful menstruation) and dyspareunia (pain during intercourse) [78]. Conducting longitudinal studies facilitates monitoring the quality of life (QoL) among individuals with endometriosis over extended periods. This methodology enables the assessment of how factors such as disease progression, treatment interventions, and various life stages influence overall well-being. Furthermore, clinical trials and intervention studies offer significant insights into the effects of different treatments—whether medical, surgical, or psychological—on QoL by employing pre- and post-treatment evaluations. Additionally, it is essential to examine indirect impacts on QoL, which may include absenteeism, diminished work productivity, and economic burdens. These effects are often assessed by utilizing tools such as the Work Productivity and Activity Impairment (WPAI) Questionnaire, which provides valuable data for analysis [79].

A critical examination of the substantial impacts of endometriosis on various aspects of quality of life (QoL) necessitates an evaluation of the methodologies employed in the existing studies, as well as an understanding of their limitations. The design of each study is paramount, and many exhibit noteworthy strengths, such as the application of validated QoL instruments, including the SF-36, WHOQOL-BREF, and condition-specific instruments like the EHP-30. Additionally, cross-sectional studies offer valuable insights into QoL at different stages of disease progression. Nevertheless, establishing causal relationships between the severity of endometriosis and QoL outcomes presents challenges. Several factors must be taken into consideration when assessing the impact of endometriosis on QoL. Variations in QoL instruments and definitions can result in heterogeneity of results, thereby affecting the comparability of findings across studies. Moreover, the recruitment of participants from clinical settings may lead to the underrepresentation of individuals without access to specialized care.

The sample size utilized in many studies also critically influences the analysis; small sample sizes often limit the generalizability of the results. Furthermore, a significant limitation in study design is the absence of multidimensional assessments that simultaneously evaluate the physical, emotional, social, and occupational impacts of endometriosis.

In summary, endometriosis profoundly impacts both the social and professional facets of a woman’s life, often leading to social isolation, strained relationships, and difficulties in career maintenance. The chronic pain, fatigue, and emotional challenges associated with this condition can disrupt daily activities and limit engagement in social and professional roles. However, effective management strategies, robust support systems, and appropriate workplace accommodations can alleviate some of these challenges, enabling women to enhance their quality of life and develop strategies to mitigate social isolation, strengthen relationships, and navigate career obstacles.

## 6. Support Strategies to Improve QoL for Women with Endometriosis

Improving the quality of life for women with endometriosis necessitates a multifaceted approach that encompasses medical and psychological support, social networks, workplace accommodations, and self-care strategies. These support mechanisms are instrumental in managing symptoms, alleviating stress, and providing essential resources to address the challenges associated with this condition [80].

### 6.1. Medical Support

It is imperative for women with endometriosis to seek healthcare providers who possess specialized expertise in the area to ensure accurate diagnosis and the formulation of effective treatment plans. Pain management typically involves the use of medications, including nonsteroidal anti-inflammatory drugs (NSAIDs), hormone therapies, and other pharmaceuticals designed to address pain effectively. In certain cases, collaboration with pain management specialists, such as physiotherapists, alongside gynecological teams is essential for comprehensive care. Furthermore, alternative therapies, including acupuncture, physical therapy, and transcutaneous electrical nerve stimulation (TENS), may serve to enhance pain relief and overall symptom management [81].

### 6.2. Psychological and Emotional Support

Access to mental health professionals, including psychologists and counselors specializing in chronic illness, is vital for assisting women in coping with the emotional and psychological ramifications of endometriosis. Therapeutic modalities, particularly cognitive behavioral therapy (CBT), have proven to be beneficial [82,83]. Establishing support groups that facilitate engagement, whether in person or online, is also crucial in providing emotional support and fostering coping strategies. Practices such as mindfulness meditation, yoga, and relaxation techniques are recognized for their efficacy in managing stress and reducing pain perception, thereby contributing to overall well-being.

### 6.3. Social and Relationship Support

The role of social and relational support is significant in enhancing the quality of life for women affected by endometriosis. Developing a robust network of friends, family, and support groups can mitigate the emotional challenges posed by the condition. Open communication with employers, colleagues, and loved ones regarding endometriosis enables improved support and necessary accommodations. Furthermore, discussions with partners regarding the impacts of endometriosis on physical and emotional intimacy are essential. Couples therapy may provide valuable assistance in addressing challenges related to intimacy and family planning [84].

### 6.4. Professional and Workplace Support

Providing educational materials about endometriosis to employers and colleagues is crucial in cultivating a supportive work environment, thus allowing women to manage their symptoms more effectively. Additionally, specialized counseling can assist in navigating professional careers and financial planning by offering guidance on managing career objectives and accessing financial assistance programs or grants to alleviate treatment costs [85].

### 6.5. Educational Resources

Accessing up-to-date information regarding the latest research and treatment options is paramount. Consulting trustworthy sources and engaging with expert groups specializing in endometriosis can enhance women’s understanding of their condition and personal needs, thereby ensuring they receive appropriate care and support for optimal health. The integration of conventional and alternative treatments tailored to individual preferences constitutes a significant aspect of achieving the best possible outcomes [86].

### 6.6. Lifestyle Modifications

Implementing lifestyle changes can markedly enhance the quality of life for women with endometriosis. Adopting an anti-inflammatory diet that emphasizes fruits, vegetables, whole grains, and omega-3 fatty acids may assist in symptom management. Seeking guidance from a nutritionist or dietitian with expertise in endometriosis can yield tailored dietary recommendations that support holistic health and wellness. Furthermore, engaging in gentle and regular physical activities, such as walking, swimming, or yoga, can contribute to pain management, stress reduction, and improvements in both physical and mental health [87].

Collectively, these support strategies empower women to effectively navigate the physical and emotional challenges associated with endometriosis, enabling them to lead fulfilling and comfortable lives. Access to comprehensive care, robust social support, and practical resources is essential for addressing the complexities inherent to this condition.

## 7. Conclusions

Endometriosis is a multifaceted and frequently debilitating condition, necessitating recognition of its physical, emotional, and social ramifications to provide comprehensive care that effectively addresses both the medical and psychological needs of patients. A thorough understanding of the impact of endometriosis and the available support methodologies is vital for enhancing the quality of patient care. The impact of endometriosis varies significantly among individuals, with the severity of symptoms differing markedly. Consequently, the treatment approach often requires customization to suit each patient’s unique circumstances. Familiarity with diverse treatment modalities, including medical interventions such as hormone therapy and surgical options like laparoscopy, ensures that the therapeutic strategy aligns with the patient’s specific concerns, whether focused on pain management, fertility preservation, or the overall quality of life.

Consequently, a multidisciplinary approach is paramount and must involve a collaboration between gynecologists, pain management specialists, fertility experts, and mental health professionals. Understanding endometriosis thoroughly aids in monitoring symptoms, identifying issues early, and re-evaluating treatment plans when needed. This approach helps prevent disease progression and addresses new challenges promptly.
